# Common bean (*Phaseolus vulgaris* L.) PvTIFY orchestrates global changes in transcript profile response to jasmonate and phosphorus deficiency

**DOI:** 10.1186/1471-2229-13-26

**Published:** 2013-02-13

**Authors:** Rosaura Aparicio-Fabre, Gabriel Guillén, Montserrat Loredo, Jesús Arellano, Oswaldo Valdés-López, Mario Ramírez, Luis P Íñiguez, Dario Panzeri, Bianca Castiglioni, Paola Cremonesi, Francesco Strozzi, Alessandra Stella, Lourdes Girard, Francesca Sparvoli, Georgina Hernández

**Affiliations:** 1 Centro de Ciencias Genómicas, Universidad Nacional Autónoma de México, Av. Universidad 1001, Mor. 62209, Cuernacaca, México; 2 Instituto de Biotecnología, Universidad Nacional Autónoma de México, Av. Universidad 1001, Mor. 62209, Cuernacaca, México; 3 Istituto di Biologia e Biotecnologia Agraria, CNR, Via Bassini 15, 20133, Milano, Italy

**Keywords:** Transcription factors, *TIFY* gene family, *Phaseolus vulgaris PvTIFY* genes, JA-signaling, Transcriptome analysis, P-starvation response

## Abstract

**Background:**

*TIFY* is a large plant-specific transcription factor gene family. A subgroup of *TIFY* genes named JAZ (Jasmonate-ZIM domain) has been identified as repressors of jasmonate (JA)-regulated transcription in *Arabidopsis* and other plants. JA signaling is involved in many aspects of plant growth/development and in defense responses to biotic and abiotic stresses. Here, we identified the TIFY genes (designated *PvTIFY*) from the legume common bean (*Phaseolus vulgaris)* and functionally characterized *PvTIFY10C* as a transcriptional regulator.

**Results:**

Nineteen genes from the *PvTIFY* gene family were identified through whole-genome sequence analysis. Most of these were induced upon methyl-JA elicitation. We selected *PvTIFY10C* as a representative JA-responsive *PvTIFY* gene for further functional analysis. Transcriptome analysis via microarray hybridization using the newly designed Bean Custom Array 90 K was performed on transgenic roots of composite plants with modulated (RNAi-silencing or over-expression) *PvTIFY10C* gene expression. Data were interpreted using Gene Ontology and MapMan adapted to common bean. Microarray differential gene expression data were validated by real-time qRT-PCR expression analysis. Comparative global gene expression analysis revealed opposite regulatory changes in processes such as RNA and protein regulation, stress responses and metabolism in *PvTIFY10C* silenced vs. over-expressing roots. These data point to transcript reprogramming (mainly repression) orchestrated by *PvTIFY10C*. In addition, we found that several *PvTIFY* genes, as well as genes from the JA biosynthetic pathway, responded to P-deficiency. Relevant P-responsive genes that participate in carbon metabolic pathways, cell wall synthesis, lipid metabolism, transport, DNA, RNA and protein regulation, and signaling were oppositely-regulated in control vs. *PvTIFY10C*-silenced roots of composite plants under P-stress. These data indicate that *PvTIFY10C* regulates, directly or indirectly, the expression of some P-responsive genes; this process could be mediated by JA-signaling.

**Conclusion:**

Our work contributes to the functional characterization of *PvTIFY* transcriptional regulators in common bean, an agronomically important legume. Members from the large *PvTIFY* gene family are important global transcriptional regulators that could participate as repressors in the JA signaling pathway. In addition, we propose that the JA-signaling pathway involving *PvTIFY* genes might play a role in regulating the plant response/adaptation to P-starvation.

## Background

Transcription factors (TF) are master-control proteins in all living cells that bind to DNA in the vicinity of target genes. TFs interact with other transcriptional regulators, including chromatin remodeling/modifying proteins, to recruit or block access of RNA polymerase to the DNA template, thus activating or repressing transcription. In plants, TFs play pivotal regulatory roles in developmental processes and responses to environmental conditions. An average of 5.7% of plant genes code for TFs, which are distributed among 62 gene families. The legumes *Medicago truncatula*, *Lotus japonicus* and soybean (*Glycine max*) each possess 1473, 1637 and 5557 TF genes, respectively [1].

Legumes are important for sustainable agriculture as they are able to form nitrogen-fixing symbioses with rhizobia and soil-nutrient scavenging symbioses with mycorrhizal fungi. Common beans (*Phaseolus vulgaris* L*.*) are the most important crop legumes for human consumption [2]. The *P. vulgaris* genome was recently sequenced and is now available in Phytozome [3,4], but the whole set of TF genes has not yet been identified. An expression platform based on real-time quantitative RT-PCR (qRT-PCR) was developed for a subset of 372 common bean TF genes and has been used to analyze their expression profiles in plants subjected to abiotic stresses such as phosphorus (P) deficiency [5,6]. Two TFs from the TIFY family were up-regulated in P-deficient common bean roots and nitrogen-fixing root nodules [5,6]. On this basis, and considering the importance of the plant-specific TIFY TF family as transcriptional regulators, in this work we analyzed the TIFY TFs in common bean.

*TIFY*, previously known as *ZIM* (Zinc-finger protein expressed in Inflorescence Meristem) [7], is a large plant-specific gene family functionally annotated as TFs. It includes 18 members in *Arabidopsis*, 20 in rice, 22 in poplar and 34 in *Glycine soja* (wild soybean) [8-10]. TIFY genes are classified into two groups depending on the presence (group I) or absence (group II) of a GATA-Zn finger domain. A sub-family of TIFY group II bearing a highly conserved Jas motif of 22 amino acids, currently named JAZ (Jasmonate ZIM-Domain), has been intensively investigated for their roles in jasmonate hormonal responses (reviewed in [11-15]).

Jasmonate (JA), an oxylipin originating from the oxidation of linolenic acid, is a plant hormone that regulates many aspects of plant growth, development and defense responses to both biotic aggressors (herbivores and necrotrophic pathogens) and abiotic stresses such as drought, UV radiation and ozone [11]. Jasmonoyl isoleucine (JA-Ile), one of the JA derivatives collectively known as jasmonates (JAs), has been identified as the active form of this hormone [16-18]. Three independent groups identified the JAZ family as repressors of JA-regulated transcription in *Arabidopsis*[17-19]. In plant cells containing low JA levels, JAZ proteins bind to and repress TFs such as MYC2 that promote transcription of JA-responsive genes. The molecular mechanism for this repression involves the NINJA/TPS (Novel Interactor for JAZ/TOPLESS) co-repressor complex [20]. JAZ-mediated repression is relieved in response to stimuli that activate JA synthesis and JA-Ile accumulation, which in turn stimulates physical interaction between JAZ and COI (Coronatine Insensitive 1), the F-box component of an SCF-type E3-ubiquitin ligase (SCF^COI1^) [21]. This interaction allows JAZ proteins to be ubiquitinated by SCF^COI1^ and subsequently degraded by the 26S proteasome. COI1 has been identified as the receptor of JA-Ile and coronatine, a bacterial elicitor phytotoxin structurally and biologically related to JA [22,23]. The functions of COI1, JAZ and MYC2 in JA signaling are analogous to those of the core components of the auxin-signaling pathway [22].

Hormone-dependent transcriptional activation has to be tightly regulated to avoid a harmful runaway response; thus, the JA pathway has an auto-regulatory mechanism with a negative feedback loop involving JAZ and MYC2. Upon degradation of JAZ repressors in response to JA, MYC2 activation induces *JAZ* gene expression, thus replenishing the JAZ pool and ensuring the formation of repressor JAZ-MYC2 complexes to limit the response after initial JA perception [18].

JA is involved in the regulation/signaling of different stress responses. *TIFY* genes from *Arabidopsis*, rice and soybean respond to abiotic stresses such as drought, high salinity, low temperature, UV radiation, ozone, bicarbonate stress, potassium deficiency, and P starvation [9-11,24-26].

Low phosphate availability represents one of the most common constraints for plant growth and crop production [27]. Plants have evolved diverse strategies to obtain adequate P under limiting conditions; these include molecular, biochemical, physiological and morphological responses such as modification of root architecture and expansion of root area [28-31]. In *Arabidopsis*, P deficiency induces a determinate root growth program in which meristematic cells have a limited number of divisions, undergo early cellular differentiation and have a gradual reduction of the cell elongation zone, resulting in a short root phenotype characterized by exhaustion of the meristem [32]. *Low phosphorus insensitive (lpi) Arabidopsis* mutants show altered root architecture in response to low P, with indeterminate root growth leading to a long primary root in P-limiting conditions [33]. Transcriptome analysis revealed that genes from the JA biosynthesis and signaling pathways were strongly down-regulated in *lpi4*; these included genes for JA biosynthesis and the *JAZ* (*TIFY*) genes *JAZ1*, *JAZ2* and *JAZ6*[26]. Common bean plants growing under P-deficiency present with higher root to shoot dry weight ratio due to arrested root growth and proliferation of lateral roots and root hairs [5]. TFs from the *TIFY* gene family are induced in roots and nodules of P-deficient plants [5,6]. Taking into account the involvement of JA signaling in the *Arabidopsis* root tip response to P-starvation [26], the crucial role of *JAZ* (*TIFY*) TF genes in the JA signaling pathway [17-19] and the induction of *TIFY* genes in P-stressed bean roots [5], we decided to analyze the possible involvement of *TIFY* and JA-signaling in the modulation of bean root architecture in response to P-starvation.

In this work, we identified genes coding for TFs from the *TIFY* gene family, hereafter termed *PvTIFY*, and examined their response to JA elicitation. We selected *PvTIFY10C* as a representative JA-responsive *PvTIFY* gene to analyze its role as a transcriptional regulator using a reverse genetic approach—RNAi-gene silencing and over-expression—and global transcriptome analysis through microarray hybridization. We used Gene Ontology [34] and MapMan [35] bioinformatic tools adapted to common bean to interpret the microarray gene expression data. *PvTIFY10C*-silenced and over-expressing roots clearly showed global reprogramming of gene expression. In addition, we investigated the possible regulatory role of PvTIFYs in the response to P starvation. Opposite regulation of gene expression in silenced vs. control and over-expressing roots under P-deficiency indicated that *PvTIFY10C* may regulate, directly or indirectly, the expression of some P-responsive genes.

Our work extends the knowledge of the role of *TIFY*s as important global transcriptional regulators involved in JA signaling and the response/adaptation to abiotic stresses such as P starvation in an agronomically important legume.

## Methods

### Identification and sequence analysis of the *Phaseolus vulgaris TIFY* gene family

**Figure 1:**
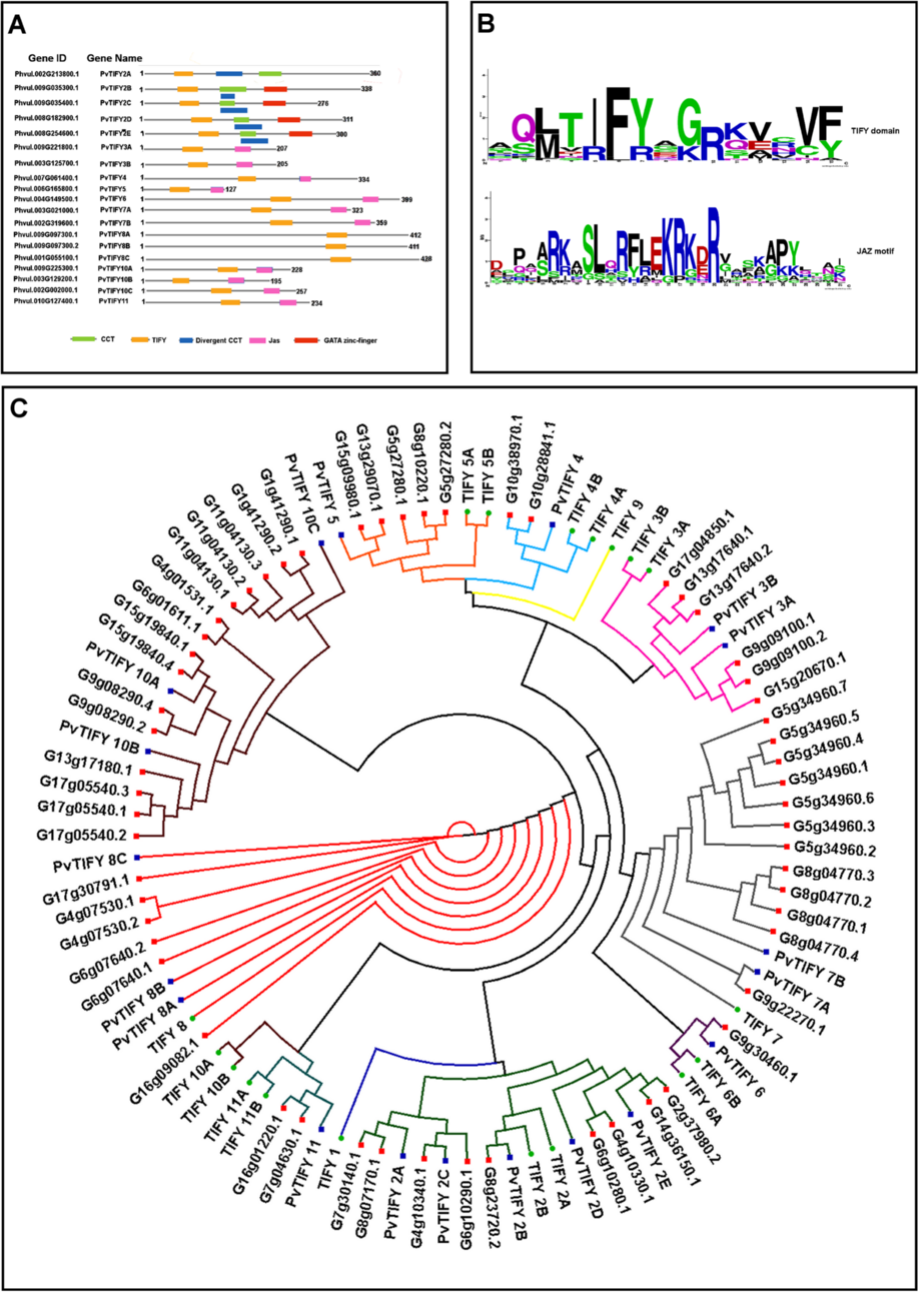
**Sequence and structural comparison of *TIFY *family members.** (**A**) Structural organization of the identified *P. vulgaris* TIFY genes. Conserved motifs: CCT, TIFY, divergent CCT, Jas and GATA zinc-finger, are depicted in colored boxes assigned as indicated at the bottom of the panel. The genes are denoted by their species (Pv) abbreviation followed by the gene name; transcript IDs [4] are also provided. (**B**) WebLogo [38] of the putative TIFY and JAS motifs constructed from appropriate subsets of the sequence alignment of 23 bean TIFY sequences with *Arabidopsis* homologs. For the Jas motif analysis the TIFY2 family, which lacks this motif, was excluded. (**C**) Phylogenetic tree of the TIFY family. Protein sequences were aligned using ClustalX. A neighbor-joining tree was constructed using the PHYLIP package from 18, 58 and 19 amino acid sequences of TIFY family members from *A. thaliana, G. max* and *P. vulgaris,* respectively. The branches are color-coded to indicate their phyletic association to the *Arabidopsis* TIFY classification. A green, red or blue dot at the end of each branch indicates genes from *A. thaliana, G. max* or *P. vulgaris*, respectively.

Gene family analysis was done using the common bean (*Phaseolus vulgaris*) and soybean (*Glycine max*) genome sequence platforms from Phytozome [3,4]. Local BLAST searches of all the proteins encoded by the common bean and soybean genomes was carried out using the HMM profile (build 2.3.2) [36] of the TIFY domain as a query. The HMM profile of the 36 amino acid-long TIFY domain (ID PF06200) was downloaded from the Pfam database [37]. The common bean and soybean *TIFY* genes identified are listed in Additional file 1. The identified genes that were duplicated genes or that showed an E-value higher than threshold (Additional file 1) were not considered for phylogenetic analysis. The identified common bean *PvTIFY* genes were named following the *Arabidopsis TIFY* family numbering system as shown in Figure 1A.

For *TIFY* sequence analysis, the sequences were input into ClustalX 1.8 [39] for joint multiple alignment. These multiple alignments were sent to the PHYLIP 3.57 package [40]. This package inputs the aligned sequences into the SEQBOOT algorithm (bootstrap sequence data sets) to create 100 data sets by bootstrap resampling. These data sets were entered into PROTDIST to generate 100 protein distance matrices. These matrices were entered into the program NEIGHBOR to produce 100 phenograms of the *TIFY* family sequences using the neighbor-joining method, followed by the creation of a majority-rule, strict consensus, unrooted tree with confidence intervals generated using CONSENSE. The resulting phylogenetic tree was displayed and edited in the SplitsTree 4 software [41].

### Plant material and growth conditions

The common bean (*Phaseolus vulgaris*) Mesoamerican cv. "Negro Jamapa 81" was used in this study. Surface-sterilized germinated seedlings were grown in a hydroponic system under controlled environmental conditions as previously described [42]. The hydroponic trays contained Franco/Munns full-nutrient solution [43] for control (C) conditions. To induce P deficiency (−P) the nutrient solution was deprived of K_2_HPO_4_. Root samples from C and − P plants were harvested at 7, 14, 21 and 25 d after planting. For methyl jasmonate (Me-JA) elicitation, plants were grown for 10 d in C media (time 0) and then 25 μM Me-JA was added to the nutrient solution for 10 min. Subsequently, the roots were washed in sterile water for 5 min and immediately transferred to a tray containing fresh C media for 12 h. For sampling, a third of each root was cut at time 0 (control), after incubation with Me-JA (10 min) and after washing off Me-JA (12 h). All root samples were immediately frozen in liquid N_2_ and preserved at −80°C until used for RNA isolation.

Common bean composite plants with transgenic roots [44] were generated as described below and grown in similar conditions to those described for un-transformed bean plants. Samples of transgenic root tissues were collected in liquid nitrogen and preserved (−80°C) until tested.

### RNA isolation

Total RNA was isolated from 1 g of frozen roots from WT plants and transgenic composite plants grown in control or stress conditions in independent experiments, as previously reported [45]. These samples were preserved at −80°C until tested. Isolated RNA preparations were used for real-time quantitative RT-PCR (qRT-PCR) and for common bean microarray hybridization as described below.

### Real-time quantitative RT-PCR (qRT-PCR)

Quantification of transcript levels was done by one-step assay using the iScript One-Step RT-PCR Kit with SYBR Green (Bio-Rad, Hercules, CA, USA). Each reaction (15 μl) contained 7.5 μl of Master Mix 2×, 100 nM forward primer, 100 nM reverse primer, 100 ng RNA template, and 0.5 μl iScript reverse transcriptase. DNase/RNase-free water was used to adjust the volume to 15 μl. Assays were run in a 96-well plate using the iQ5 Real-Time PCR Detection System and iQ5 Optical System Software (Bio-Rad). The thermal cycler settings were as follows: 10 min at 50°C (cDNA synthesis), 5 min at 95°C (iScript reverse transcriptase inactivation), followed by 40 cycles for PCR cycling and detection of 30 s at 55°C. Each assay had a melt curve analysis consisting of 80 cycles of 1 min at 95°C, 1 min at 55°C, and 10 s at 55°C, increasing each by 0.5°C per cycle. For each reaction, a product between 75 and 150 bp could be visualized on an agarose gel. Each assay included at least two no-template controls, in which RNA was substituted with DNase/RNase-free water. No amplification was detected for the no-template controls. Quantification was based on a cycle threshold (C_T_) value, with the expression level of each gene normalized to the C_T_ value of the house-keeping elongation factor 1 (*PvEF1)* gene. The sequences of oligonucleotide primers used in qRT-PCR are shown in Additional file 2. Statistical analysis was done using Bartlett’s test to determine whether groups exhibited similar variance. The average normalized values of gene expression were compared with one-way ANOVA followed by Tukey’s multiple comparison test when significant difference (*p* < 0.05) of means occurred.

We determined whether the qRT-PCR assay for *PvTIFY10C* and *PvEF1* was optimized. Assays were run using duplicates of four-fold serial dilutions of RNA from bean roots to generate a standard curve by plotting the log of the starting quantity of RNA against the C_T_ value obtained during amplification of each dilution. The amount of RNA per well ranged from 50 to 800 ng; a negative control with distilled water was also included. The equation of the linear regression line and the coefficient of determination (R^2^) were calculated to evaluate the quality of the assay. To determine the specificity of the primers used for *PvTIFY10C* and *PvEF1*, after completion of the amplification reaction, a melt curve was generated by increasing the temperature in small increments and monitoring the fluorescent signal at each step following the protocol: 1 min at 95°C, 1 min at 55°C, 10 sec at 55°C (80 cycles, increasing by 0.5°C each cycle). The data shown in Additional file 3 indicated that the qRT-PCR assay was optimal and that the primers used were specific.

### Cloning of full-length cDNA and promoter region from *PvTIFY10C*

The *PvTIFY10C* full-length cDNA sequence was cloned based on partial EST sequences assigned to TC34164 [46], as previously reported [45]. Two primers were designed for *PvTIFY10C* PCR gene amplification by 5′-rapid amplification of cDNA ends (RACE) (GSP5: TGG CCG GAA AAT TCA GAG TAC TCC GAC G) and 3′ RACE (GSP3: TGA CAA TCT TTT ATG GTG GAC AAG TTG TTG TG). 5′ and 3′ RACE was performed using the SMART-RACE-cDNA Amplification kit (Clontech Laboratories, Inc., Mountain View, CA, USA).

The sequence of *PvTIFY10C* full-length cDNA was submitted to GenBank (accession no. JX645706).

A 3,561 bp fragment from the 5′-end (promoter) region of *PvTIFY10C (*Phvul.002G002000.1) was cloned using the Universal Genome Walker kit (BD Biosciences, San Jose, CA, USA), following the manufacturer’s protocol. Genomic DNA (100 ng/μl) from bean roots was digested with the blunt-end restriction enzymes DraI, EcoRV, PvuII, and StuI and subsequently ligated to the provided adaptor linkers. Nested PCR with a 94°C initial denaturation was then performed using gene-specific primers homologous to the *PvTIFY10C* coding region (5′-GAGTACTCCGAC GAGCTGGACATGATG and 5′-ACTGCAAGTTTGA GAGAAGCTGGACTTC) and adaptor primers (5′-GTAATACGACTCACTATAGGGC and 5′-ACTATAGGG CACGCGTGGT) from the Advantage 2 PCR kit (BD Biosciences). The cycle parameters were as follows: seven cycles of 94°C for 25 s and 72°C for 3 min, followed by 32 cycles of 94°C for 25 s and 67°C for 3 min, with a final extension at 67°C for 7 min. The DNA products were then purified, subcloned into the pCR2.1-TOPO (Invitrogen, Carlsbad, CA, USA) vector and sequenced.

### Sub-cellular protein localization

For protein localization assays, the pMCD83 vector [47] was used for N-terminal translational fusion to *PvTIFY10C*. To generate this construct, a 774 bp fragment from the *PvTIFY10C* coding sequence was amplified using gene-specific forward (5′-CACCATGTCCAGCTCGTCG GAGTACTCTG) and reverse (5′-CCAGAACTTAGA GAAGGGTTCCG) primers. The amplified fragment was cloned into the pENTR/SD/D-TOPO vector (Invitrogen) and sequenced. The resulting pENTR-PvTIFY10C plasmid was recombined into the pMCD83 binary vector.

The plasmids pMDC83 and pPvTIFY:GFP were introduced into *Agrobacterium tumefaciens* LB4404 by electroporation and these strains were used to transform onion (*Allium cepa*) epidermal cells. *A. tumefaciens* strains were cultured overnight in 2 ml LB broth plus 50 mg/ml kanamycin, pelleted and re-suspended (OD_600_ = 1) in 0.5× MS media supplemented with 10 mM MgCl_2_ and 100 μM acetosyringone. A fragment of onion epidermal layer was pierced with a needle and infiltrated by introducing it into a tube containing the *A. tumefaciens* cell suspension. These were cultured for 12 h at 25°C before examining the fluorescence with a Zeiss Axiovert 200 M inverted microscope.

### Plasmid construction

To obtain a *PvTIFY10C* over-expression construct, we first constructed the pTDTO vector. This was derived from the pTDT-DC-RNAi vector previously reported [45]. The pTDT-RNAi Gateway cassette was removed by digesting pTDT-DC-RNAi with XbaI and the resulting linear vector was dephosphorylated. A fragment containing a multi-cloning site (MCS) and the *loxP*Gm3 interposon was amplified from a modified pJMS2 plasmid [48] using  specific forward (5′-GAGGTCTAGACGGTCTCGAGAAGCTGGATCCATAACTTCGTATAA) and reverse (5′-TAATCTAGAACCCGGGCCCTATATTTGGATCCAATTGCAATGATC) primers. Restriction sites for XbaI, XhoI and BamHI in the forward primer and for XbaI, SmaI and BamHI in the reverse primer are underlined. The PCR product was cut with XbaI and cloned into the XbaI sites of the linearized pTDT-DC-RNAi. The gentamycin (Gm) cassette, from Gm-resistant selected clones, was removed by BamHI digestion and the resulting vector (pTDTO) was sequenced. The pTDTO vector has a MCS, with XhoI, SmaI and BamHI unique sites, between the 35S CaMV promoter and the NOS terminator, as well as the reporter tdTomato gene from the pRSET-BtdTomato vector [49].

To generate the over-expression construct, a fragment (1018 bp) unique to *PvTIFY10C* containing the whole cDNA sequence (775 bp) was obtained by PCR. For amplification, cDNA from common bean roots was used together with the specific forward (5′-TCTCGAGT CACCGAATACTTGTGTTC-3′) and reverse (5′-ATG GATCCAAATAAAGGGGTAACAAGAAAC-3′) primers. Restriction sites for XhoI and BamHI in the forward and reverse primers, respectively, are underlined. This PCR product was cloned by T-A annealing into pCR 2.1-TOPO (Invitrogen) and analyzed by sequencing. The 1018 bp XhoI-BamHI fragment from pCR 2.1-TOPO was cloned into the XhoI-BamHI sites in pTDTO. The resulting pPvTIFY-OE plasmid (Additional file 4) was analyzed by sequencing and used to over-express the *PvTIFY10C* gene in common bean transgenic roots.

To obtain a pPvTIFY-RNAi construct (Additional file 4), we used the pTDT-DC-RNAi vector previously reported [45]. A fragment (374 bp) unique to the *PvTIFY10C* coding sequence was amplified using gene-specific forward: 5′-CAAAGAACCTGACAGCCATGGATTTG and caccTIFY reverse: 5′- CACCGGCCTGGATGATGCTTGAGAGTG primers. The amplified fragment was cloned into the pENTR/SD/D-TOPO vector (Invitrogen) and sequenced. The resulting pENTR-*PvTIFY10C* plasmid was recombined into the pTDT-DC-RNAi binary vector. The correct orientation was confirmed by PCR using the WRKY-5: 5′-GCAGAGGAGGAGAAGCTTCTAG and WRKY-3: 5′-CTTCTCCAACCACAGGAATTCATC primers.

The empty pTDTO vector (used as a control; hereafter termed EV) and the resulting pPvTIFY-OE and pPvTIFY-RNAi plasmids (Additional file 4) were introduced by electroporation into *A. rhizogenes* K599, which was then used for plant transformation.

### Plant transformation and production of composite plants

The protocol used was based on that of Estrada-Navarrete et al. [44], with the following modifications. *P. vulgaris* seeds were surface sterilized and germinated in disposable Petri dishes with filter paper soaked in sterile water. After two days the seed coats were removed and on the third day plantlets were infected at the cotyledonary node region with the *A. rhizogenes* K599 strain carrying one of the constructs described above (EV, pPvTIFY-OE or pPvTIFY-RNAi). Infected plantlets were transferred to sterile assay tubes containing 15.0 ml Falcon plastic tubes filled with Franco/Munns nutrient solution [43], which provided support and a humid, sterile environment. The assay tubes containing the plantlets were covered with plastic bags to preserve the humidity required for hairy root development. Fourteen days after infection, putative transgenic hairy roots were confirmed by checking for the presence of red fluorescence resulting from the expression of the tdTomato reporter gene using an epifluorescence stereomicroscope. The original root system and non-fluorescent primordia and hairy roots were excised, to avoid root chimeras, and the selected composite plants carrying only fluorescent roots were transferred to a hydroponic system (described above) for growth. After 7–10 days of growth adaptation in hydroponics, the composite plants were transferred to different growth conditions (as described above for non-transformed plants) depending on the experiment (*i.e.* P-deficiency, Me-JA elicitation).

### Bean Custom Array 90 K design and hybridization

A bean microarray was printed using the CombiMatrix platform with a custom 90 K array layout at the Plant Functional Genomic Center of the University of Verona, Italy. To define the layout, an in-house bioinformatics pipeline was created to collect, compare and filter bean and soybean RNA sequences available from the bean gene index [46] version 3.0 (21,497 total unique sequences) and NCBI UniGene build 38.0 (http://www.ncbi.nlm.nih.gob/unigene; 33,001 reference soybean genes). Since the bean sequences could not be considered a complete set in terms of representation of genes and transcripts, these were taken as references and the soybean UniGenes were added to have a larger dataset available. Duplicated sequences were then removed, using a homology search pipeline with BlastN, to define a minimally redundant dataset of transcripts. After this step, all sequences in the dataset were processed to design probes of 35–40 nucleotides according to CombiMatrix parameters. These probes were subsequently filtered to avoid cross-hybridization across the different targets. The final layout contained 18,867 unique bean sequence probes and 11,205 soybean UniGene probes, along with positive and negative controls, for a total of 30,150 different features available on the microarray. Each probe was printed in triplicate to ensure the presence of internal replicates and to have a good statistical representation of each transcript on the array. The microarray was designated the Bean Custom Array 90 K.

RNA was isolated from transgenic roots of composite plants that were transformed with EV, pPvTIFY-RNAi or pPvTIFY-OE. Total RNA (1 μg) was used as a template to synthesize antisense RNA (aRNA) with Cy5-ULS using the RNA Amplification and Labeling Kit from CombiMatrix (ampULSe, Kreatech Biotechnology) according to manufacturer’s instructions. Prehybridization was performed by incubating the arrays with prehybridization solution (6× SSPE, 0.05% Tween-20, 20 mM EDTA, 5× Denhardt’s solution, 100 ng/μl Salmon Sperm DNA, 0.05% SDS) for 30 min at 45°C. Labeled aRNA (4 μg) was fragmented by incubation with 5 μl of fragmentation solution (200 mM Tris Acetate pH 8.1, 500 mM KOAc, 150 mM MgOAc) for 20 min at 95°C.

Hybridization was performed at 45°C for 16 h in hybridization solution (6× SSPE, 0.05% Tween-20, 20 mM EDTA, 25% DiFormamide, 100 ng/μl Salmon Sperm DNA, 0.04% SDS). After hybridization and washing, the microarray was dipped in imaging solution, covered with LifterSlip™, and then scanned using a GenePix 4000B microarray scanner (Axon) and the accompanying acquisition software (CombiMatrix Microarray Imager Software). Multiple scans at different PMTs were provided for each hybridization.

### Microarray data analysis

The data discussed in this publication have been deposited in NCBI’s Gene Expression Omnibus [50] and are accessible through GEO Series accession number GSE40935 (http://www.ncbi.nlm.nih.gov/geo/query/acc.cgi?acc=GSE40935).

The raw intensity data were first processed using the Combimatrix Microarray Imager software. This program allows visual inspection of the entire microarray slide and is used to check the quality of each spot and, if needed, to perform corrections where possible (*i.e.* for dust or scratches on the surface). Intensity data were then exported into the Feature and Probe format of Combimatrix, where the actual raw intensity per probe and per spot is stored. Data were loaded into R and analyzed using the Limma package [51]. The median value of each spot on the array was considered and the probes were filtered to remove quality and factory controls. Within-array probe replicates were defined as technical replicates, by calculating the mean intensity across the different probes. The probe intensity values of each biological condition were normalized using the quantile function of Limma. The values present in the expression matrix were transformed into log_2_ and a design matrix was defined to describe the biological samples. The expression matrix was used to fit a linear model using the design matrix and the functions of Limma. A set of contrast matrices were defined to describe the comparisons among samples in the experiment and a second linear fitting was performed for each contrast. The data were then error corrected using the Bayesian functions of Limma and a list of differentially expressed genes was generated for each contrast, after correcting for multiple testing using the Benjamin-Hochberg method and setting 0.05 as the adjusted *P*-value cutoff.

We used Gene Ontology (GO) [34] and MapMan [35] bioinformatics-based approaches for analyses aimed to interpret the biological significance of gene expression data. The ESTs corresponding to the microarray probe sets were organized in functional categories according to GO guidelines (34). We assigned at least one GO term to 23,499 probe sets. The Fisher’s exact test [52] was applied to determine which GO categories were statistically over-represented within each set of differentially expressed ESTs analyzed (*p* < 0.05, corrected by Bonferroni adjustment). A second approach for expression data analysis was based on the MapMan software version 3.5.1 (http://gabi.rzpd.de/projects/MapMan/) [35]. To extend MapMan to common bean, a *Phaseolus vulgaris* map was developed and uploaded to MapMan. The change in expression ratio of each gene was calculated as the log_2_-fold change to generate the MapMan experimental file. The MapMan software was used to visualize the amplitudes of the changes in expression of individual genes in diagrams of metabolic pathways or cellular processes.

## Results and discussion

### Identification and phylogenetic analysis of *P. vulgaris TIFY* gene family

*TIFY* is a large plant-specific gene family, so far characterized in only a few plant species [8-10,12,14]. To identify all putative TIFY domain-containing proteins in common bean, an HMM profile [36] of the TIFY domain was identified. The TIFY domain was used as a query for a BLAST search against the common bean genome [4]. This search identified 19 unique *TIFY* gene family members from common bean (Figure 1A). These genes were named following the existing numbering system of the *Arabidopsis TIFY* family in the phylogenetic tree described below. Members from the *TIFY* sub-families 2, 3, 4, 5, 6, 7, 8, 10 and 11 were identified in the common bean genome (Figure 1A).

The predicted amino acid sequence of each PvTIFY protein was analyzed to identify conserved putative functional domains [8,13,14]. All 19 PvTIFY proteins contained the TIFY domain (Figure 1A) [8]. Fifteen amino acids were used to create a TIFY domain logo [38] for the common bean proteins analyzed. The TIFY consensus motif (TIF[F/Y]XG) [8,13] was present in all of the common bean proteins analyzed (Figure 1B). Five PvTIFY proteins from the PvTIFY2 sub-family (PvTIFY2A, 2B, 2C, 2D and 2E) contained CCT and divergent CCT domains, while four of these (PvTIFY2B, 2C, 2D and 2E) also contained a DNA-binding GATA zinc-finger domain (Figure 1A). Eleven PvTIFY proteins (PvTIFY3A, 3B, 4, 5, 6, 7A, 7B, 10A, 10B, 10C and 11) contained the Jas motif located near the C-terminus (Figure 1A) [11,13,14]. The sequences used to create the Jas motif logo [38] were 31 amino acids in length (Figure 1B). The Jas motif sequence was very similar to that of *Arabidopsis*, including relevant features such as 2 basic amino acids in the N-terminal end and a conserved PY at the C-terminal end [13].

To analyze the evolutionary relationships of the *PvTIFY* genes, a phylogenetic tree was generated using the sequence alignments of TIFY proteins from common bean, soybean and *Arabidopsis* (Figure 1C). As we did with common bean, a local BLAST search of all proteins encoded by the soybean genome was carried out using the HMM profile and 58 unique TIFY gene family members were identified (Additional file 1). Though Zhu et al. reported 34 soybean (*G. max*) *TIFY* genes [10], we identified a larger number and used these to generate the phylogenetic tree (Figure 1C). Five proteins from the PvTIFY2 sub-family clustered together with nine soybean *TIFY* genes; according to the *Arabidopsis* TIFY classification [8] these belonged to group I. The other 14 PvTIFY proteins, without the GATA-zinc finger domain, belonged to the second major group (group II). These clustered together with 49 TIFY proteins from soybean. Group II contained all the JAZ proteins in *Arabidopsis* and putative JAZ homologs in common bean (Figure 1C).

The number of *TIFY* genes identified in common bean was similar to that reported for the dicots *Arabidopsis* and poplar and for the monocot rice [8-10,16]. *PvTIFY* genes from subfamilies 1 and 9 were not identified but their existence cannot be ruled out in view of the incomplete genome sequence currently available [4]. Eleven members of the common bean *PvTIFY* gene family can be considered *JAZ* orthologs. These contained the TIFY and Jas domains (Figure 1A, 1B). The TIFY domain mediates homo- and heteromeric interactions between the *Arabidopsis* TIFY proteins and interacts with the NINJA protein from the NINJA/TPL repressor complex. The Jas domain mediates hormone-dependent JAZ degradation by the SCF^COI1^/26S proteasome pathway in *Arabidopsis*[18,20]. Basic amino acids from the N-terminal end of the Jas domain are required for COI1 interaction with JAZ1 and JAZ9 in *Arabidopsis*[21]; similar basic amino acids (RK) were identified in the common bean JAZ orthologs (Figure 1B). We propose that the common bean JAZ proteins participate in the JA signaling pathway as negative regulators in a similar mechanism to that demonstrated in *Arabidopsis*[11-15]. However, further functional analysis is required to demonstrate the function of the PvTIFY proteins.

### *PvTIFY10C* gene structure, promoter sequence analysis and protein sub-cellular localization

For further analysis of the *PvTIFY* genes as transcriptional regulators we chose *PvTIFY10C*, encoded by the Phvul.002G002000.1 locus, as a representative JA-responsive *PvTIFY* gene. Similarly to rice (9), the *P. vulgaris PvTIFY10* sub-family contained three members. *PvTIFY10A* and *PvTIFY10B* had the highest similarity to *Arabidopsis TIFY10A* (or *JAZ1*) and *TIFY10B* (or *JAZ2*), respectively, and the third gene was named *PvTIFY10C*. Our previous work had shown that *PvTIFY10C* (TC34164) [46] is up-regulated in P-stressed common bean roots and nodules, which will be discussed below [5,6].

**Figure 2:**
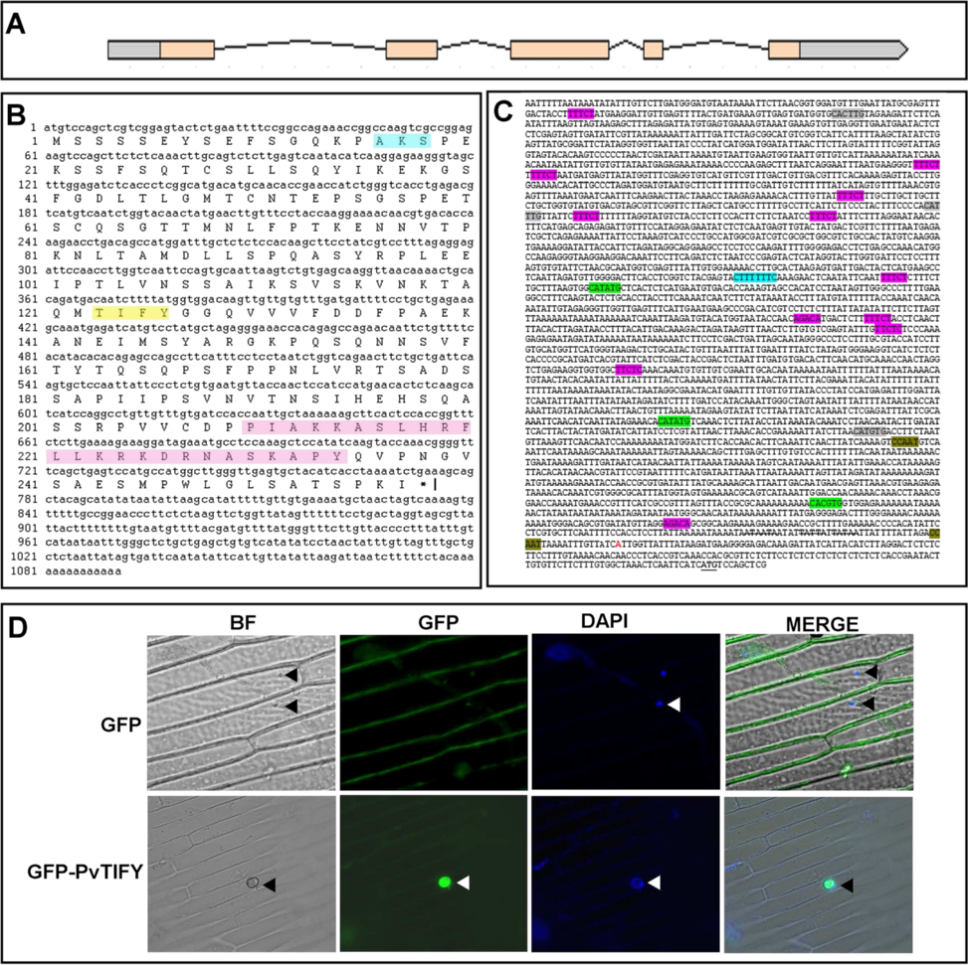
**Structural organization and cellular localization of *PvTIFY10C*.** (**A**) *PvTIFY10C* gene structure. Exon regions are indicated with salmon-colored boxes, introns with black lines, and 5^′^ and 3^′^ UTRs with gray boxes. (**B**) Deduced amino acid sequence of *PvTIFY10C*. A predicted sumoylation site is shaded in blue. The TIFY domain is shaded in yellow. The Jas motive is shaded in pink. (**C**) The promoter region includes important regulatory *cis*-elements: CCAAT motifs (brown) at −15 and −713, G-boxes (CATATG; green) at −267, -911 and −1923, E-boxes (CANNTG; gray) at −798, 2474 and 3189, HUD (Hormone Up at Dawn; pink) elements at −41, -1304, -1563, -1642, -1655, -1947, -2785, -2788, -2791 and −3240, and a JA-responsive element (CTTTTNTC) at −1973. (**D**) *PvTIFY10C* is located in the nucleus. Onion epidermal cells were transiently transformed with a 35S:PvTIFY-GFP (GFP-TIFY) construct or with an empty vector (GFP). Epifluorescence (GFP, DAPI and MERGE) and bright-field (BF) images were captured of onion epidermal cells. Arrowheads indicate nuclei.

Sequence analysis revealed that *PvTIFY10C* was organized into five exons and four introns (Figure 2A). We obtained a full-length *PvTIFY10C* c-DNA clone (1.24 kb, accession number JX645706) from the Mesoamerican “Negro Jamapa 81” cultivar (see Methods). It contained a 775-bp open reading frame that encoded a 277 amino acid protein with 57% similarity to *Arabidopsis* TIFY10A. The TIFY domain, Jas motif and a putative sumoylation site were identified from the deduced amino acid sequence (Figure 2B).

To get insight into the regulation of *PvTIFY10C* gene expression we analyzed a 3,561-bp sequence from the 5′ region (Figure 2C). A putative site for transcription initiation (YYCAYYYY) was identified, as were three putative TATA-boxes within the −32 to −50 region and two CCAAT-boxes at −15 and −713; these are essential for TF binding and RNA polymerase II dependent transcription [53]. Other putative regulatory *cis*-elements identified in the *PvTIFY10C* promoter sequence included a JA-responsive element (CTTTTNTC) at −1973 to −1980 [54] and three G-boxes (CATATG and CACGTG variants). G-boxes reside in the promoters of many genes that are switched on in response to various stimuli after binding of the so-called G-box binding factors, which include bZIP proteins. Representative examples of G-box–containing promoters are those that respond to light, anaerobiosis and hormones such as JA, abscisic acid and ethylene [54,55]. Three E-boxes were also identified; these participate in complex regulatory circuits related to the circadian clock and low temperature stimuli [56]. Finally, *cis*-elements known as Hormone Up at Dawn (HUD)-type E-boxes were identified; these elements bind MP and ARF TFs and are involved in the response to auxin and brassinosteroids [57].

The sequence analysis of the *PvTIFY10C* gene promoter suggested strong regulation of the expression of this gene by plant phytohormones. This included not only JA-responsive gene expression, which is crucial for the feedback regulatory loop in the JA signaling pathway [9,17-19], but might also include cross-talk responses mediated by related cues such as ethylene, auxins, and brassinosteroids. In this regard, Grunewald et al. reported that the *Arabidopsis TIFY10A (JAZ1)* gene is an early auxin-responsive gene and that auxin gene induction is independent of the JA signaling pathway [58]. Chacón-López et al. reported that JA and ethylene act synergistically to trigger the meristem exhaustion process characteristic of the *Arabidopsis* root tip response to P-deprivation that includes induction of *JAZ* genes [26].

*Arabidopsis* TIFY proteins from group II lack a known DNA-binding domain but are localized to the nucleus, a pre-requisite for a protein to function as a transcriptional regulator [8]. We analyzed the sub-cellular localization of PvTIFY10C, which belongs to group II (Figure 1). For this, we constructed a fusion protein containing the *PvTIFY10C* open reading frame (Figure 2B) at the C-terminus and GFP at the N-terminus to serve as a marker. This construct was transiently expressed after *A. tumefaciens* delivery to onion cells. The GFP-PvTIFY10C protein was localized to the nucleus of onion cells, in contrast to GFP used as control, which was observed in the cytosol (Figure 2D). The sumoylation site detected in the deduced PvTIFY10C amino acid sequence (Figure 2B) might play a role in directing the protein to the nucleus [59]. Thus, the data supported our proposal of PvTIFY10C as a transcriptional regulator.

### Response of *PvTIFY* to JAs elicitation in roots from wild type plants and in transgenic roots showing *PvTIFY10C* gene silencing or over-expression

**Figure 3:**
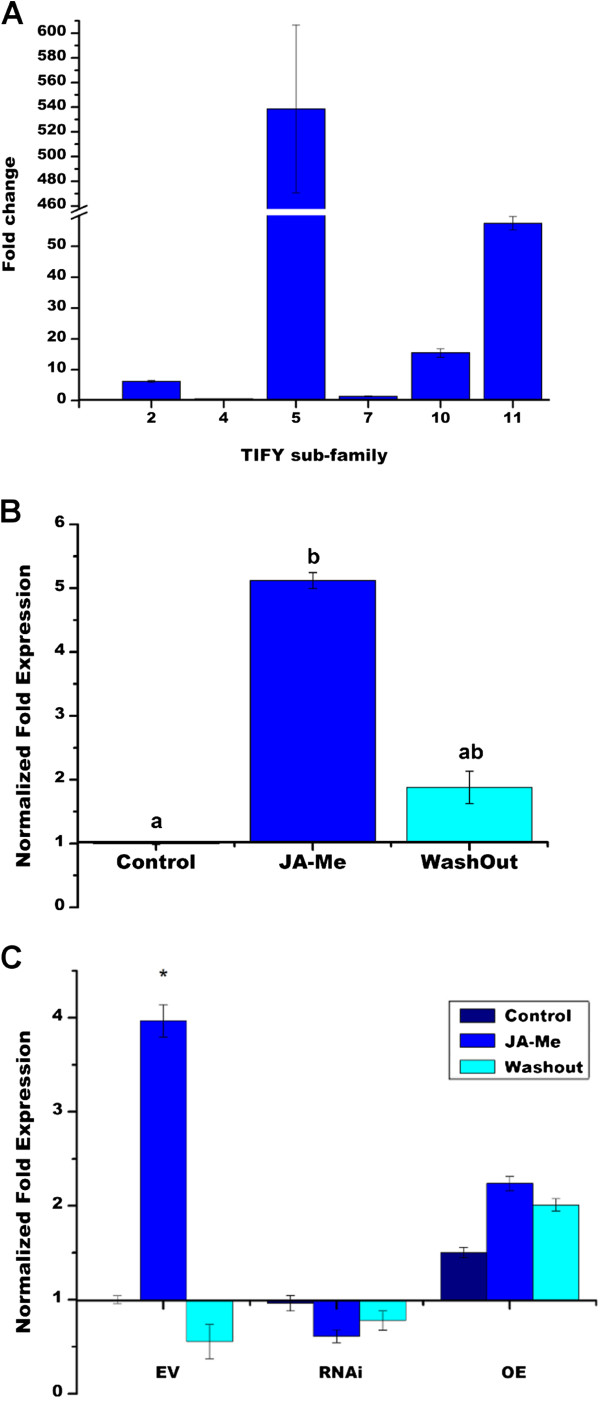
**Expression of *PvTIFY *genes upon JA elicitation.** (**A**) *n*-Fold expression of *PvTIFY* genes from different subfamilies in common bean roots incubated with Me-JA. (**B**–**C**) Normalized *n*-fold expression of the *PvTIFY10C* gene in wild type (**B**) and transgenic (**C**) bean roots without Me-JA (time 0, control media; dark blue) or incubated with Me-JA for 30 min (blue). After incubation, Me-JA was depleted from the nutrient solution and 12 h later gene expression was determined (turquoise). Values are normalized to the value from the control conditions (without Me-JA), which was set to 1. Transgenic roots derived from composite bean plants transformed with pPvTIFY-RNAi or pPvTIFY-OE are indicated. Values represent the average of three biological replicates. Asterisks or different letters represent significantly different means according to statistical analysis (*p* < 0.05).

In *Arabidopsis*, most *JAZ* genes from the *TIFY* gene family are rapidly induced by Jas; this feedback loop presumably replenishes the repressors of JA response to avoid runaway stress metabolism [17-19]. We assessed whether *PvTIFY* genes from different sub-families responded to JA-Me elicitation. *Arabidopsis* and soybean TIFY genes of the same sub-family show sequence similarity and a similar response to elicitors [10,17,18,60]. In the common bean PvTIFY genes we identified, members of the same subfamily also showed high DNA sequence conservation (30–99%) and could have similar regulation and functions. We analyzed the response to JA-Me of the identified *PvTIFY* gene sub-families (Figure 1A) by qRT-PCR assay using a specific primer pair for each gene sub-family based on a DNA sequence that was highly conserved or identical among the different members. *PvTIFY* genes from sub-families 2, 4 and 7 showed reduced induction whereas *PvTIFY5, 10* and *11* showed high induction upon Me-JA elicitation (Figure 3A).

Gene expression responses to hormones are usually quick and transient; therefore, we verified the early response of *PvTIFY10C* to Me-JA (after 30 min) and also the expected recovery after washing out the elicitor. After a short exposure to JA-Me the *PvTIFY10C* transcript level, as determined by qRT-PCR, increased up to 5-fold. Subsequently, the plant nutrient solution was deprived of Me-JA and the *PvTIFY10C* transcript level recovered, after 12 h, to almost basal levels (Figure 3B). These results confirmed the high and transient response of *PvTIFY10C* to Me-JA in common bean roots.

The response of common bean roots to JAs elicitation was also assessed in transgenic roots of composite plants with modulated expression—silencing or over-expression—of *PvTIFY10C*. For this, and further analyses, we used a protocol for the generation of composite plants with transformed roots using *A. rhizogenes*-mediated transformation that has been established as an alternative for stable transformation, especially for recalcitrant species such as *P. vulgaris*[44]. This approach is suitable for carrying out functional genomics in common bean in conjunction with RNAi-silencing technology [45].

To achieve gene silencing we constructed a pPvTIFY-RNAi plasmid that contained inverted repeats of a *PvTIFY10C* fragment and for over-expression we constructed a pPvTIFY-OE plasmid with the full-length *PvTIFY10C* cDNA. The 35SCaMV constitutive promoter directed the expression of both transgenes. The expression of *PvTIFY-RNAi* and *PvTIFY-OE* in putative transgenic roots was verified by qRT-PCR using specific primers.

Throughout this work we obtained around 100 composite bean plants with transgenic roots bearing one of the constructs (EV, PvTIFY-RNAi or PvTIFY-OE). Each transgenic root results from a different transformation event and therefore each individual root transformed with pPvTIFY-RNAi or pPvTIFY-OE will show a specific degree of gene silencing or over-expression, respectively. The figure presented as Additional file 4 illustrates this phenomenon; it shows the normalized *n*-fold expression of *PvTIFY10C* in 25 representative individual transgenic roots from different composite plants with PvTIFY-RNAi or PvTIFY-OE. Though a high variation in the degree of gene silencing and over-expression was observed in this sample of transgenic roots, the expected tendency in modulation of *PvTIFY10C* expression was confirmed. The average *PvTIFY10C* expression ratio in silenced compared with control EV transgenic roots was 0.27 (± 0.9), and in over-expressing roots was 2 (± 8.32).

To determine whether the *PvTIFY10C* RNAi-silencing or over-expression affected the JA response observed in common bean roots, we performed a similar experiment to that described for non-transformed plants (Figure 3B) and analyzed the early and transient response to Me-JA in transgenic roots. The response observed in control transgenic roots was very similar to that of non-transformed roots; control EV roots from composite plants exposed to JA-Me showed an increase in *PvTIFY10C* expression that decreased after the elicitor was washed out (Figure 3C). However, the transient response of *PvTIFY10C* to Me-JA elicitation was altered in silenced or over-expressing roots. In silenced transgenic roots, *PvTIFY10C* showed low/basal expression when plants were grown in control media, and the level was similar both upon subsequent Me-JA elicitation and after the elicitor was washed out (Figure 3C). The altered response to Me-JA elicitation in PvTIFY-RNAi roots may indicate that the expressed inverted-repeat of RNAi-PvTIFY promotes intense degradation of the *PvTIFY10C* mRNA even in the presence of the JA-Me inducer. The expected gene over-expression was observed in the PvTIFY-OE roots, which showed *ca.* two-fold higher *PvTIFY10C* transcript levels in control conditions compared with EV roots. A constitutive strong promoter (35SCaMV) was used for *PvTIFY10C* over-expression, and correspondingly enhanced levels of *PvTIFY10C* transcript were observed in all conditions tested (control, Me-JA elicitation and wash-out) for PvTIFY-OE roots (Figure 3C). PvTIFY-OE roots showed similar over-expression levels irrespective of the elicitation or depletion of JA-Me (Figure 3C). These results indicated that the modulation of *PvTIFY10C* expression in common bean roots altered their response to JAs; *PvTIFY10C* silenced or over-expressing transgenic roots were insensitive to this elicitor.

### Microarray analysis of transgenic roots with RNAi-silencing or over-expression of *PvTIFY10C*

Our rationale for further analysis was that the modulation (silencing or over-expression) of a transcriptional regulator results in reprogramming of the transcript profile; therefore, we performed transcriptome analysis through a microarray-hybridization approach of transgenic roots from composite bean plants that showed modulated *PvTIFY10C* gene expression. We designed the Bean Custom Array 90 K, which included a 30 K unigene set from common bean (*ca.* 18,000 *P. vulgaris* ESTs and *ca*. 11,000 non-redundant soybean (*Glycine max*) ESTs). The microarray data discussed in this work have been deposited in NCBI’s Gene Expression Omnibus [50] and are accessible through GEO Series accession number GSE40935 (http://www.ncbi.nlm.nih.gov/geo/query/acc.cgi?acc=GSE40935). To assess the reliability of the microarray data, the normalized median signal intensities resulting from the samples were compared across two replicates and three repetitions of each biological sample. A high correlation coefficient of 0.82–0.98 was observed among the samples, indicating low variability between replicates.

**Figure 4:**
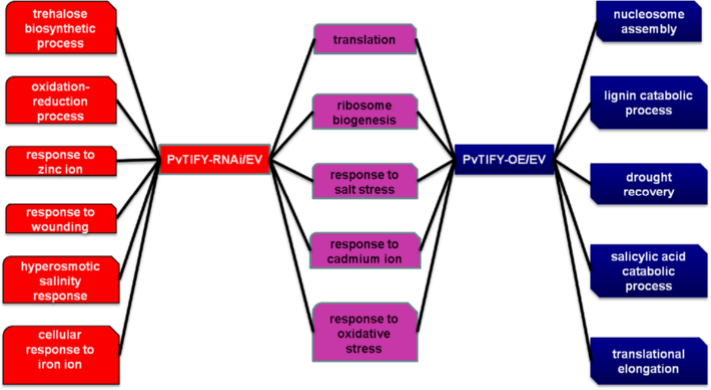
**Significantly over-represented biological processes according to Gene Ontology in the responsive EST sets from PvTIFY-RNAi (red) and PvTIFY-OE transgenic roots (blue).** Biological processes over-represented in both EST sets are shown in pink.

To interpret the biological significance of the microarray data our first analysis was based on Gene Ontology (GO) [34] bioinformatic approaches. Functional categories were assigned to 23,499 probe sets from the microarray, according to GO guidelines. Statistical analysis was performed to identify the GO categories that were over-represented in each set of responsive ESTs from PvTIFY-silenced (PvTIFY-RNAi) and over-expressing roots (PvTIFY-OE), compared with control roots transformed with an empty vector (EV). As shown in Figure 4, sixteen biological processes (GO categories) were over-represented in both EST sets; five of these were shared by PvTIFY-RNAi and PvTIFY-OE roots. The majority of over-represented biological processes were related to plant stress responses. These included responses to oxidative stress, metals (Fe, Zn, Cd), wounding, salinity and drought, and also signaling processes involved in the stress response such as salicylic acid and trehalose (Figure 4). The latter is in agreement with the participation of *PvTIFY* genes in the JA signaling pathway, which is strongly related to stress responses [11,25]. The silencing or over-expression of the *PvTIFY10C* TF changed the transcription profile of genes participating in JA-mediated plant stress responses.

To obtain a more complete interpretation of the gene expression data, we also used the MapMan software tool [35], customized for common bean, to analyze the data generated from our microarrays. Our rationale was that the genes controlled by *PvTIFY10C* (at the transcriptional level) would be oppositely-regulated in roots with decreased vs. increased levels of this TF. To this end, we performed comparative transcriptome analysis of PvTIFY-RNAi and PvTIFY-OE roots with respect to control EV roots, aiming to identify the set of genes regulated by *PvTIFY10C*. The microarray data from PvTIFY-RNAi/EV and PvTIFY-OE/EV were statistically analyzed using MapMan (corrected *P* values < 0.05; threshold fold change [log_2_] of 0.1 or −0.1). PvTIFY-RNAi roots showed a majority of down-regulated ESTs (734 vs. 312 up-regulated ESTs) while PvTIFY-OE roots showed 561 up-regulated and 625 down-regulated ESTs (Additional file 5).

**Figure 5:**
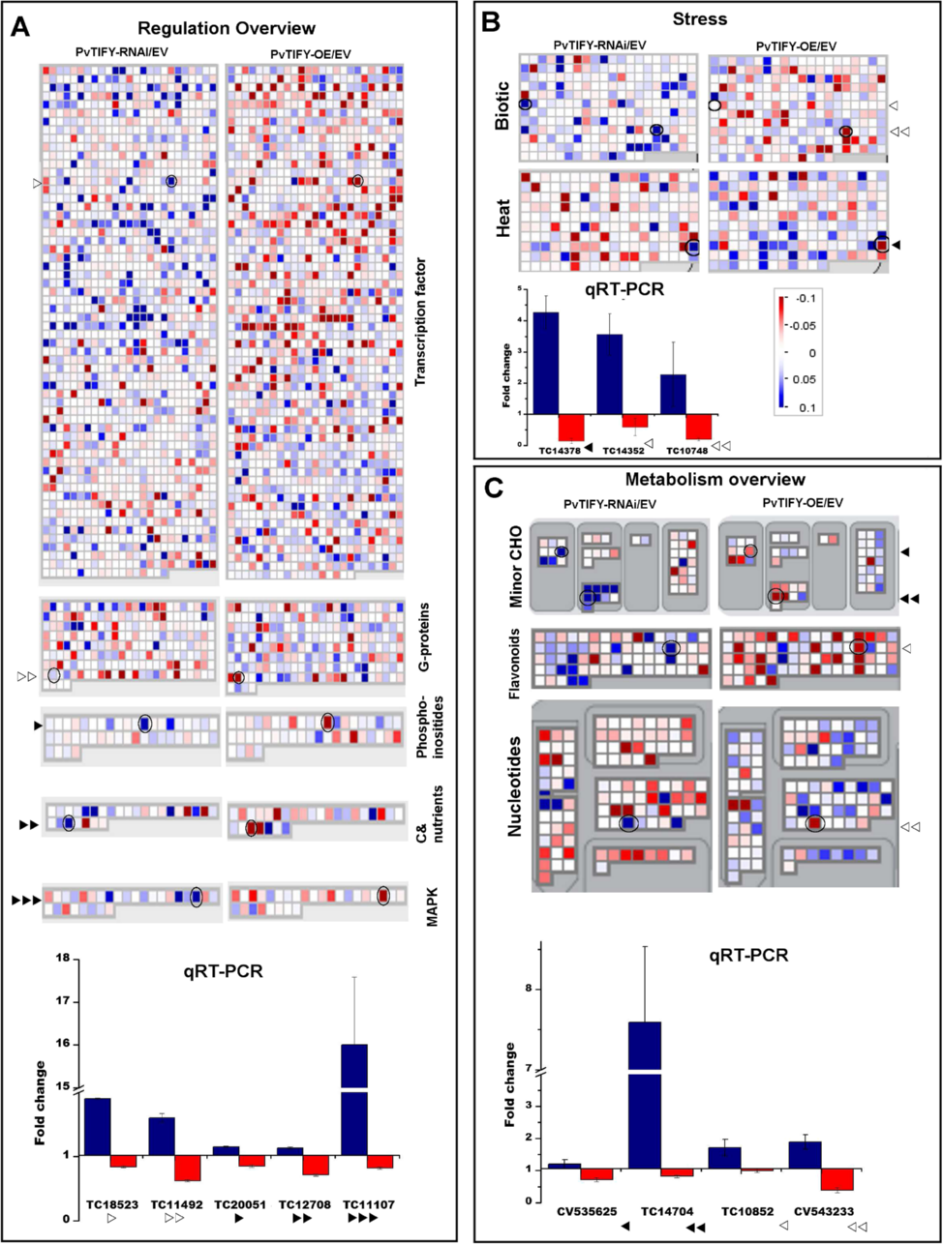
**Transcript profiles of PvTIFY-RNAi and PvTIFY-OE transgenic roots compared with EV (control) transgenic roots.** MapMan maps of processes belonging to the regulation overview (**A**), stress response (**B**) and metabolism overview (**C**) categories are shown. In each map, the left panel shows PvTIFY-RNAi/EV expression ratios and the right panel shows PvTIFY-OE/EV, as indicated. Up- or down-regulated ESTs are false color-coded with increasing blue or red, respectively, saturating at amplitude of 0.1 (log_2_ value) as indicated in the bar from panel **B**. ESTs with no significant change in amplitude are shown in white. Expression ratios of selected ESTs obtained from microarrays (maps) were validated by qRT-PCR analysis. Normalized data from qRT-PCR gene expression assays are shown in bar-graphs at the bottom of panels **A**, **B** and **C**. The ESTs selected for qRT-PCR analysis correspond to the circled squares from each map and are indicated with black and white arrowheads. The arrowheads in the maps correspond to those in the bar-graphs. The ESTs ID [46] are indicated in the x-axes of the bar-graphs. In each pair of bars, the blue bar corresponds to the PvTIFY-RNAi/EV expression ratio (left in the maps) and the red bar corresponds to the PvTIFY-OE/EV expression ratio (right in the maps). The annotations for the selected ESTs are mentioned in the text.

Figure 5 shows example maps depicting cell processes or pathways used to compare gene expression in *PvTIFY10C* silenced vs. over-expressing roots. The modulation of *PvTIFY10C* resulted in drastic changes in the transcript profiles of different processes in the regulation overview (Figure 5A); silenced roots showed 179 up-regulated and 85 down-regulated ESTs, while over-expressing roots showed 54 up-regulated and 221 down-regulated ESTs (Additional file 5). The regulation overview includes BIN 27: RNA regulation of transcription (TF) and BIN 30: signaling with G-proteins, phosphoinositides, signaling in sugar and nutrient physiology and MAPK, both of which clearly showed an abundance of oppositely-regulated ESTs in *PvTIFY10C*-silenced (up-regulation) vs. over-expressing roots (Figure 5A). To validate the differential expression data from the microarray analysis, we selected one TC from each category of the regulation overview to determine its transcript level through qRT-PCR. The selected TCs [46] were: TC18523 (annotated as C2H2 zinc finger TF family), TC11492 (Rho-GTPase-activating protein-related), TC20051 (phosphatidylinositol 3- and 4-kinase family protein), TC12708 (phosphate-responsive protein) and TC11107 (MAP kinase/kinase/protein kinase). The expression levels of these genes determined by qRT-PCR confirmed the expression results obtained with microarray analysis regarding the up-regulation in PvTIFY-RNAi roots and down-regulation in PvTIFY-OE roots (Figure 5A). The variations in expression level values between the microarray and qRT-PCR data may be related to the different sensitivities of the two technologies.

JA signaling involving *TIFY* TF genes plays an essential role in stress responses including defense against insects and microbial pathogens (biotic stress), as well as responses to abiotic stresses such as drought, UV radiation and ozone [11,25]. Figure 5B depicts maps of biotic stress and heat abiotic stress responsive genes that belong to BIN 20. Opposite differential stress response gene expression was observed in PvTIFY-RNAi (33 up-regulated and 18 down-regulated ESTs) vs. PvTIFY-OE roots (10 up-regulated and 23 down-regulated ESTs, Additional file 5). Two TCs from the biotic stress map were selected for validation of their expression levels by qRT-PCR: TC14352 (annotated as a putative secretory protein) and TC14378 (a PR-protein from the resistance responsive family), and one TC was selected from the heat abiotic stress map: TC10748 (annotated as a DNAJ heat shock N-terminal domain containing protein). Plant responses to stress include the synthesis of secondary metabolites, such as flavonoids. The map of flavonoid genes, corresponding to BIN 16, is shown in Figure 5C within the metabolic overview. From this group, TC10852 (annotated as a cinnamoyl-CoA reductase family protein) was selected for qRT-PCR expression validation. Again, the expression results from qRT-PCR confirmed those from the microarrays regarding up-regulation of the selected stress response TCs in *PvTIFY10C*-silenced roots and down-regulation in over-expressing roots (Figures 5B and 5C).

JA also regulates diverse aspects of plant growth and development that involve regulation of central metabolic pathways. We performed MapMan analysis of differentially expressed genes from different categories of the metabolism overview. Figure 5C shows, as an example, maps of minor CHO and nucleotide metabolism. Opposite differential gene expression was also observed in the metabolism overview category for PvTIFY-RNAi (97 up-regulated and 108 down-regulated ESTs) vs. PvTIFY-OE roots (54 up-regulated and 102 down-regulated ESTs, Additional file 5). qRT-PCR expression analysis of selected ESTs—CV535625 (annotated as a putative imbibition protein) and TC14704 (trehalose phosphatase/synthase) from the minor CHO metabolism (BIN 3.2.3) and CV543233 (annotated as an inorganic diphosphatase/pyrophosphatase) from the nucleotide metabolism phosphotransfer pyrophosphatases (BIN 23.4)—confirmed the expression values obtained from the microarray.

**Figure 6:**
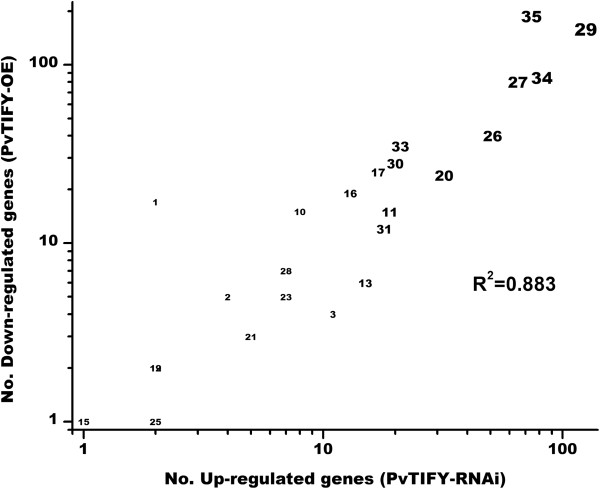
**Correlation between the genes up-regulated in PvTIFY-RNAi and down-regulated in PvTIFY-OE transgenic roots.** ESTs significantly up-regulated in PvTIFY-RNAi/EV or down-regulated PvTIFY/EV expression ratios were considered. Data were extracted from the MapMan [37] Venn diagram workflow visualization (threshold fold change [log_2_] of 0.1 or −0.1). The bubble chart was constructed using the row numbers of functional categories (BINs). The size of the numbers denotes number of ESTs showing a significance response at the selected amplitude (≥ 0.1).

Based on the MapMan analysis of selected cell processes (depicted in Figure 5) that showed opposite transcriptional effects in roots with contrasting *PvTIFY10C* gene expression, we performed a general analysis by determining the correlation of up-regulated ESTs in PvTIFY-RNAi roots with down-regulated ESTs in PvTIFY-OE roots and *vice versa*. Figure 6 shows the correlation among up-regulated ESTs in PvTIFY-RNAi roots and down-regulated ESTs in PvTIFY-OE with a correlation coefficient of 0.833. For this correlation, the gene categories corresponding to different BINs from MapMan [35] and the number of genes from each BIN were considered. Most of the represented BINs included a large number of genes (10–200) that were up-regulated in silenced roots and down-regulated in over-expressing roots. Besides the “not assigned” BIN 35, the BINs that included the highest number of oppositely-regulated genes in *PvTIFY10C*-silenced roots (up-regulated) vs. over-expressing roots were: proteins (BIN 29), which included the sub-BINs synthesis, targeting, degradation, post-translational modification; RNA (BIN 27), which included processing, transcription, regulation of transcription; transport (BIN 34), which included extra- and intra-cellular transport processes; development (BIN 33), which included storage proteins; miscellaneous enzyme families (BIN 26), which included gluco-, galacto- and mannosidases, nitrilases, glutathione S transferases, peroxidases; signaling (BIN 30), which included receptor kinases, MAP kinases, G-proteins; and stress (BIN 20), which included biotic and different abiotic stresses (Figure 6). The cell processes shown in Figure 5 were included in those BINs with large numbers of oppositely-regulated genes as shown in Figure 6.

The transcript profile reprogramming observed in PvTIFY-RNAi *vs*. PvTIFY-OE roots from our MapMan analysis of microarray data strongly supports the role of *PvTIFY10C* as a global transcriptional regulator. This TF could regulate, directly or indirectly, the transcription of genes involved in relevant processes such as regulation at the RNA and protein levels as well as different signaling pathways, responses to stress, development and pathways of the primary metabolism (Figures 5 and 6). The majority of differentially expressed genes were up-regulated in *PvTIFY10C*-silenced roots and showed opposite regulation in over-expressing roots (Figures 5 and 6), thus indicating a main role for this TF as a repressor, either directly, as has been shown for *Arabidopsis* JA-regulated transcription [17-19], or indirectly through interaction with other transcriptional regulators.

### *PvTIFY* transcriptional regulation in the response to P-deficiency

Low P availability is a major constraint for plant growth [27]. It has been demonstrated that at least five TFs regulate the *Arabidopsis* response to P-deficiency at the level of transcription; PHR1 (from the MYB family), WRKY75, ZAT6, BHLH32 and MYB62 [61-65]. In previous studies, we identified some 50 TFs that responded to P-deficiency in common bean, including PvTIFY TFs [5,6]. Through reverse genetics we demonstrated the role of PvPHR1 in P-deficiency signaling in common bean roots [45]. In addition, JA signaling has been implicated in the *Arabidopsis* root tip response to P-starvation [26]; gene expression and physiological analyses of the *lpi4* mutant revealed that JAZ (TIFY family) genes and JA biosynthesis genes were down-regulated. These observations and characterization of physiological/phenotypic responses in *lpi4 vs.* WT plants indicated that changes in redox status, mediated by JA and ethylene, play an important role in the primary root meristem exhaustion process triggered by P-starvation [26]. In this work, we explored the possible involvement of *PvTIFY* in the transcriptional regulation of the bean root response to P-starvation through transcriptome analysis.

**Figure 7:**
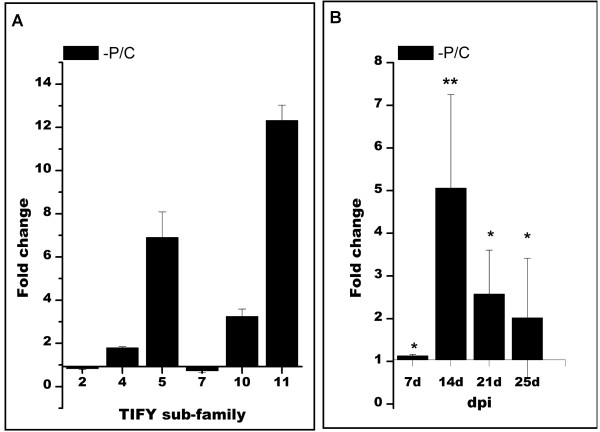
**Expression of *PvTIFY *genes in response to P-deficiency.***n*-Fold expression of *PvTIFY* genes from different subfamilies (**A**) and the *PvTIFY10C* gene (**B**) in common bean roots of plants grown in P-deficient conditions (−P) compared with roots from C plants (full-nutrient conditions). **A** Expression of *PvTIFY* genes was determined in roots from plants grown for 25 days. **B***PvTIFY10C* expression was determined in roots at different stages of development, as indicated (d = days after planting). Values represent the average of three biological replicates. Asterisks represent significantly different means compared with the control conditions, according to statistical analysis (*p* < 0.05).

We assessed the response of *PvTIFY* genes from different sub-families to –P through qRT-PCR expression analysis (Figure 7A). Similarly to the analysis of *PvTIFY* gene responses to Me-JA shown in Figure 3A, each gene sub-family’s response to –P was assayed based on the assumption that the high similarity of members within a given sub-family indicates similar gene regulation and function [10,17,18,60]. *PvTIFY* gene expression was determined in the roots of common bean plants grown for 25 d in –P conditions (Figure 7A). The *PvTIFY* genes from sub-families 4, 5, 10 and 11 were induced in P-deficient bean roots, while genes from *PvTIFY2* and *7* were slightly repressed (Figure 7A). These results were in agreement with the induction of TC34164 (encoded by *PvTIFY10C)* in − P bean roots of 21-day-old plants grown in pots with vermiculite under low P conditions [5]. Comparison of the data presented in Figures 3A and 7A indicated a similar response for each *PvTIFY* gene subfamily to the different elicitors (Me-JA and –P). Sub-families 5, 10 and 11 showed a strong response to both elicitors; *PvTIFY5* showed the highest response to Me-JA and *PvTIFY11* to –P (Figures 3A and 7A), thus indicating the importance of these PvTIFY sub-families in responses to environmental cues. PvTIFY genes from subfamilies 2 and 7 showed weaker responses, with a tendency to be repressed in the –P treatment (Figure 7A). *Arabidopsis TIFY5* (*JAZ7, JAZ8*), *TIFY10* (*JAZ1, JAZ2*) and *TIFY11* (*JAZ5, JAZ6*) were highly induced by JA elicitation [17] and *TIFY10* and *11* were also highly induced upon pathogen challenge [60]. In soybean, both *G. soja* and *G. max Gs/GmTIFY10* and *11* genes were highly induced in response to bicarbonate stress [10].

We then analyzed the *PvTIFY10C* gene expression in P-stressed bean roots at different developmental stages. This experiment was conducted under the same plant growth conditions used throughout this work, hydroponics with nutrient media deprived of P, which differed from the conditions we used previously [5]. An increase in *PvTIFY10C* expression level was observed in − P roots at both earlier (7–14 d) and later (21–25 d) developmental stages (Figure 7B).

Based on our microarray data, we analyzed whether genes encoding enzymes for JA biosynthesis were differentially expressed in − P control (EV) roots. ESTs annotated as lipoxygenase LOX1 and LOX2, allene oxide cyclase 2 (AOC2) and 12-oxophytodienoate reductase (OPR2), which catalyze the first, third and fourth steps of the JA biosynthetic pathway, respectively, showed a slight up-regulation in P-deficient common bean roots. While other transcriptome analyses of *Arabidopsis* roots from plants under P-starvation did not detect significant induction of JA biosynthetic genes [66-68], Chacón-López et al. reported the induction of these genes after analyzing global gene expression exclusively in the root tip, the part of the root that senses P-starvation [26]. Our analysis in common bean used the whole root system, so higher induction of the JA biosynthetic genes might be detected in the root tip.

To investigate whether *PvTIFY10C* acts as a transcriptional regulator of the response to P stress, we analyzed the transcript profile of bean roots under P-deficiency to detect − P-responsive genes. We hypothesized that genes that are transcriptionally regulated—directly or indirectly—by *PvTIFY10C*, would show opposite expression patterns in silenced vs. control (transformed with EV) roots from composite plants growing under − P conditions. *PvTIFY10C* would act as a transcriptional activator of genes that showed induction in control roots and repression in silenced roots and conversely, would act as a transcriptional repressor of genes that showed down-regulation in control and over-expressing roots and induction in silenced roots under P-deficiency. We also analyzed gene expression responses to P-deficiency in transgenic roots over-expressing *PvTIFY10C*; we assumed that genes that are regulated by *PvTIFY10C* would show a similar trend, up- or down-regulation, as the control roots, although perhaps with increased transcript levels.

MapMan statistical analysis (corrected *P* values < 0.05; threshold fold change [log_2_] of 0.1 or −0.1) of microarray data from EV − P/C and PvTIFY-RNAi –P/C revealed a total of 98 ESTs that were up-regulated in control and down-regulated in silenced roots; 91% of these ESTs were also up-regulated in over-expressing roots (Additional file 6). Another 148 ESTs were down-regulated in control and up-regulated in silenced roots; 83% of these were also down-regulated in over-expressing roots (Additional file 6). Both sets included genes from different categories known to be related to P-deficiency responses in common bean and other plants [5,6,66-72].

The most abundant categories of genes that were up-regulated in control and over-expressing − P roots and down-regulated in silenced − P roots were those related to central metabolism (BINs 2 and 3: major and minor CHO, BIN 4: glycolysis, BIN 5: fermentation, BIN 6: gluconeogenesis/glyoxylate cycle, BIN 8: TCA/organic acid transformations, BIN 13: amino acid metabolism, BIN 23: nucleotide metabolism), stress/defense (BIN 16: secondary metabolism, BIN 20: stress, BIN 22: polyamine synthesis, BIN 26: miscellaneous enzyme activities), RNA (BIN 27), DNA (BIN 28) or protein (BIN 29) regulation, and transport (BIN 34). Genes from these categories are considered relevant to plant responses/adaptation to P-starvation [5,6,66-72]. Table 1 shows examples of genes that were up-regulated both in control and over-expressing roots, though only half of them showed higher expression in over-expressing roots, indicating that transcriptional changes were more evident in silenced roots. Several pathways of central metabolism are activated under P-deficiency, these mainly include alternative metabolic routes that use pyrophosphate and prevent excessive P utilization. We found that genes encoding enzymes such as phosphoenolpyruvate carboxylase and malate dehydrogenase were induced in –P common bean roots (Table 1); these are key genes that participate in alternative metabolic routes up-regulated under P-stress [71]. We propose that the expression of these genes is controlled by *PvTIFY10C* (Table 1). Pathways of carbon metabolism, such as those shown in Table 1, feed the TCA cycle and participate in organic acid biosynthesis; organic acid root exudation to increase P acquisition from the rhizosphere is an important and widespread P-stress response in plants [73,74]. Proteins that are important for P acquisition and turnover such as phosphatases and transporters were also found to be induced in control and over-expressing –P roots and down-regulated in *PvTIFY10C*-silenced roots (Table 1). The complex responses of plants to P-stress require major changes in signaling/regulation processes that occur at different levels and transcriptome analyses in different plants have identified a plethora of putative signaling and regulatory genes that could be involved in P-stress signaling [66-72]. We found several genes that were up-regulated in control and over-expressing P-stressed roots that might be related to regulation at the DNA, RNA and protein levels (Table 1, Additional file 6). These included TFs from gene families that were characterized as − P-responsive in common bean roots [6]. We propose that *PvTIFY10C* acts as a negative regulator of the genes that were repressed in − P control and PvTIFY-OE bean roots and showed opposite regulation in PvTIFY-RNAi roots, such as those participating in carbon metabolic pathways, phosphate turnover, transport and regulation listed in Table 1 and Additional file 6.

**Table 1:** Selected P-responsive genes identified by microarray analysis

			**Normalized fold change (log 2) -P/C**		
**Gene**	**BinName**	**ID a**	**EV**	**PvTIFY-RNAi**	**PvTIFY-OE**
**Induced in control roots and repressed in PvTIFY-RNAi roots**					
Cell wall invertase	Major CHO	TC8333	0.132	−0.076	0.196
Seed imbibition, hydrolase	Minor CHO	EC997013, CV535625, EC911359	0.158	−0.053	0.160
Phosphoenolpyruvate carboxylase	Gluconeogenesis/ glyoxylate cycle	TC12305, FE897651, FE711062	0.140	−0.120	0.069
Malic enzyme	TCA / org. Transformation	TC19528	0.120	−0.038	0.122
Alanine-glyoxylate transaminase	Amino acid metabolism	EC911309, CV537351, TC15378	0.112	−0.095	0.189
Inorganic diphosphatase/ pyrophosphatase	Nucleotide metabolism	TC9801	0.102	−0.021	0.099
Signal transducer/ triacylglycerol lipase	Stress	TC13450	0.108	−0.067	0.113
Cold regulated 413 plasma membrane 1	Stress	TC17916	0.164	−0.012	0.151
Trypsin and protease inhibitor family protein	Stress	TC10263	0.142	−0.010	0.413
Cytochrome P450 /CYP705A2)	Miscellaneous enzyme families	TC17341	0.149	−0.024	0.125
Glutathione S-transferase	Miscellaneous enzyme families	TC14044	0.199	−0.023	0.375
inositol-polyphosphate 5-phosphatase	DNA	TC17029, TC18885	0.145	−0.002	0.064
CCAAT-binding transcription factor (CBF-B/NF-YA) family protein	RNA	TC11100	0.102	−0.036	0.057
AP2 domain-containing TF	RNA	TC17374	0.103	−0.046	0.123
Homeobox protein 6	RNA	TC18879	0.120	−0.023	0.058
Protein phosphatase 2C, putative	Protein	TC8583	0.110	−0.045	0.045
Protein kinase 2A	Protein	TC8739	0.113	−0.016	0.086
ATPase, coupled to transmembrane movement of substances	Transport	TC15570, TC9579	0.120	−0.028	0.153
Putative phosphate transporter 1 (PHO1)	Transport	CV543807	0.105	−0.015	0.022
Hypersensitive-induced response protein	Transport	TC10794	0.145	−0.031	0.181
**Repressed in control roots and induced in PvTIFY-RNAi roots**					
Fasciclin-like arabinogalactan protein, putative	Cell wall	TC8476	−0.095	0.180	−0.042
Xyloglucan endotransglucosylase/hydrolase 9	Cell wall	TC12404	−0.102	0.047	−0.057
Acetoacetyl-coa thiolase	Lipid metabolism	TC9630	−0.101	0.064	−0.152
Lipid-transfer protein, non-specific	Lipid metabolism	FE898308	−0.109	0.005	−0.025
delta-8 sphingolipid desaturase	Lipid metabolism	TC15181	−0.050	0.127	−0.118
Ovule/fiber cell elongation protein	Hormone metabolism	TC18895	−0.105	0.108	−0.142
GAST1 Protein homolog 4	Hormone metabolism	TC18314	−0.143	0.146	−0.097
Histone H4	DNA	TC14696	−0.101	0.088	−0.234
Histone H3	DNA	TC13898	−0.106	0.099	−0.186
ATHB13; DNA binding / transcription factor	RNA	TC15258	−0.122	0.047	−0.125
MYB, transcription factor	RNA	BQ481439	−0.187	0.017	−0.022
DNA binding / transcription factor	RNA	TC15305	−0.102	0.002	−0.069
Y14 RNA binding protein	RNA	CV538348	−0.101	0.022	−0.007
Remorin family protein	RNA	TC18087	−0.103	0.007	−0.012
Eukaryotic translation initiation factor 4 F, putative	Protein	TC13072	−0.100	0.004	−0.102
60S ribosomal protein L14 (RPL14B) |	Protein	TC14174, TC17981, TC12005, TC10014, TC12381	−0.095	0.130	−0.397
Receptor-like protein kinase-related	Signaling	CV532262	−0.116	0.004	−0.077
RKL1 (Receptor-like kinase 1)	Signaling	TC11321	−0.107	0.105	−0.234
RHO-Like GTP binding protein 4	Signaling	TC18636	−0.124	0.039	−0.057
Calreticulin 2	Signaling	TC16682	−0.004	0.107	−0.167

a. From bean gene index [46], ESTs encoded by the same gene present in the microarray and with similar differential expression are shown.

The most abundant categories of genes that were down-regulated in control and over-expressing − P roots and up-regulated in silenced roots were those related to the cell wall (BIN 10), amino acid metabolism and protein synthesis (BINs 13 and 29), and regulation/signaling (BINs 27, 28, and 21). Table 1 shows − P repressed genes from categories that could be important for the response to this stress; transcriptomic analyses in different plant species have shown down-regulation responses in genes from similar categories [5,6,66-72]. Several genes involved in regulation at the DNA, RNA and protein levels, as well as in signaling pathways that include hormones, were repressed in control and over-expressing –P roots; these genes are shown in Table 1 and might be relevant for the adaptation to P stress and could be negatively regulated by *PvTIFY10C*. Repression of genes participating in cell wall and protein synthesis might be relevant for the modification of root architecture, a characteristic response in P-starved roots [29-32]. Another characteristic response of plant cells to P-deficiency is the turnover of membrane lipids that results in replacement of membrane phospholipids with galacto- or sulfo-lipids. Accordingly, several lipid metabolism genes were found to be repressed in control and over-expressing − P bean roots (Table 1). We propose that *PvTIFY10C* acts as a positive regulator of the genes that were repressed in − P roots and showed opposite regulation in PvTIFY-RNAi roots, such as those listed in Table 1 and Additional file 6.

## Conclusions

The plant-specific TIFY family encoding transcriptional regulators involved in JA signaling is largely uncharacterized in plants from the legume family. In this work, we identified the *PvTIFY* genes from *P. vulgaris* (common bean), which included 19 members distributed in 9 sub-families. Phylogenetic analysis showed similarities among *Arabidopsis*, *G. max* and *P. vulgaris* TIFY genes. *PvTIFY* genes from group II have consensus TIFY and Jas domains and can be considered *Arabidopsis JAZ* orthologs. The *PvTIFY* genes from several sub-families responded to Me-JA elicitation.

Functional characterization of the *PvTIFY10C* transcriptional regulator, chosen as representative of the *PvTIFY* JA-responsive genes that localized to the nucleus, was performed through transcriptome analysis via microarray hybridization. The Bean Custom Array 90 K, originally designed by our group, was suitable for transcriptome analysis. This was combined with a reverse genetic approach; RNAi gene silencing and gene over-expression in transgenic roots of composite plants [44,45]. Transgenic roots with modulated expression of *PvTIFY10C* were insensitive to Me-JA. GO-based microarray analyses evidenced transcriptional changes in biological processes related to plant stress responses. Microarray analysis through the MapMan software led us to conclude that *PvTIFY10C* orchestrates global changes in the transcript profile, which showed opposite regulation in silenced roots (mainly gene induction) vs. over-expressing roots (mainly gene repression). *PvTIFY10C*, and perhaps other *PvTIFY* genes, is proposed to function as a repressor in JA-regulated transcription in a similar manner to that described for *Arabidopsis*[17-19].

The involvement of JA signaling in the *Arabidopsis* root tip response to P-starvation has been documented, and changes in redox status, mediated by JA and ethylene, play an important role in the primary root meristem exhaustion process triggered by P-starvation [26]. In this work, we present evidence of the response of several *PvTIFY* genes to P-starvation in common bean roots. Promoter sequence analysis of *PvTIFY10C* revealed several *cis*-regulatory elements that respond to JA and other phytohormones such as auxins and brassinosteroids, something that may be related to the *PvTIFY* regulation of root architecture upon P-starvation. In addition, we observed a slight induction of JA-biosynthetic genes in roots exposed to P-stress. Transcript profiles in control vs. *PvTIFY10C*-silenced roots led us to conclude that *PvTIFY10C*, directly or indirectly, regulates the response of several P-responsive genes that could be mediated by JA and other hormone signaling.

Our work has contributed to the functional characterization of *PvTIFY* transcriptional regulators in common bean, an agronomically important legume.

## Abbreviations

TF: Transcription factor;qRT-PCR: Real-time quantitative reverse transcriptase-polymerase chain reaction;JA: Jasmonate;Me-JA: Jasmonate methyl ester;−P: Phosphorus deficiency;EST: Expressed sequence tag;TC: Tentative consensus

## Competing interests

The authors declare that they have no competing interests.

## Authors’ contributions

RA-F, GG and GH coordinated the work and participated in analyzing results. GG and RA-F participated in sequence and phylogenetic analyses, and Mapman analysis of microarray data. FSp, AS and FSt participated in microarray design and printing. RA-F, ML, JA, MR, OVL, LG, GG, DP, BC, PC and FSp participated in the different experimental approaches used, such as: plant growth, plant transformation, determination of gene expression, plasmid construction, protein cellular localization, microarray hybridization and densitometry. GG and FSp participated in creating the common bean MapMan mapping file. FSt and AS performed statistical analyses of microarray data. GH, RA-F, GG, LG participated in drafting the manuscript. GH conceived and designed the work. All authors read and approved the final manuscript.

## Supplementary Material

Additional file 1 **TIFY genes from common bean and soybean.** A table listing the TIFY genes identified through HMM profile analysis of the common bean and soybean genome sequences from Phytozome [3,4]. Click here for file

Additional file 2 **Primers used for qRT-PCR gene expression analysis.** A table listing primers used for qRT-PCR validation of selected genes including *PvTIFY* genes from different subfamilies and genes differentially expressed in PvTIFY-RNAi and PvTIFY-OE transgenic roots. Click here for file

Additional file 3 **Quality and specificity of qRT-PCR assays.** A figure showing the standard curve (log starting quantity of RNA vs. Ct; upper panel) and dissociation curve (lower panel) of qRT-PCR assays of *PvTIFY10C* and the *PvEF1* housekeeping gene used for normalization. Click here for file

Additional file 4 **Modulation of *PvTIFY10C *gene expression in transgenic roots.** A figure showing diagrams representing the pPvTIFY-OE and pPvTIFY-RNAi plasmids used for *PvTIFY10C* over-expression and gene silencing, respectively. A graph showing the levels of *PvTIFY10C* expression in transgenic roots is also presented. Each bar represents the *PvTIFY10C* transcript level, determined by qRT-PCR, in an individual transgenic root resulting from a different transformation event with pPvTIFY-RNAi or pPvTIFY-OE. Click here for file

Additional file 5 **Differentially-regulated genes in TIFY-RNAi or TIFY-OE vs. EV transgenic roots.** A table listing microarray expression data of ESTs that were up-regulated in PvTIFY-RNAi (sheet 1), down-regulated in PvTIFY-RNAi (sheet 2), up-regulated in PvTIFY-OE (sheet 3) or down-regulated in PvTIFY-OE (sheet 4) compared with control (EV) transgenic roots. Composite plants were grown in C conditions. Data were extracted from the MapMan [4] Venn diagram workflow (threshold fold change [log_2_] of 0.1 or −0.1). Click here for file

Additional file 6 **P-deficiency responsive genes that showed opposite regulation in control (EV) vs. PvTIFY-RNAi transgenic roots.** A table listing microarray expression data of ESTs that were up-regulated in EV and down- regulated in PvTIFY-RNAi roots (sheet 1) or down-regulated in EV and up-regulated in PvTIFY-RNAi roots (sheet 2). Data were extracted from the MapMan Venn diagram workflow (threshold fold change [log_2_] of 0.1 or −0.1). Selected genes from these lists are shown in Table 1. Click here for file
