# Supplementary figures and images for: Common bean (Phaseolus vulgaris L.) PvTIFY orchestrates global changes in transcript profile response to jasmonate and phosphorus deficiency
Source: BMC Plant Biol. 2013 Feb 13;13:26. doi: 10.1186/1471-2229-13-26 (PMC3621168; doi:10.1186/1471-2229-13-26)

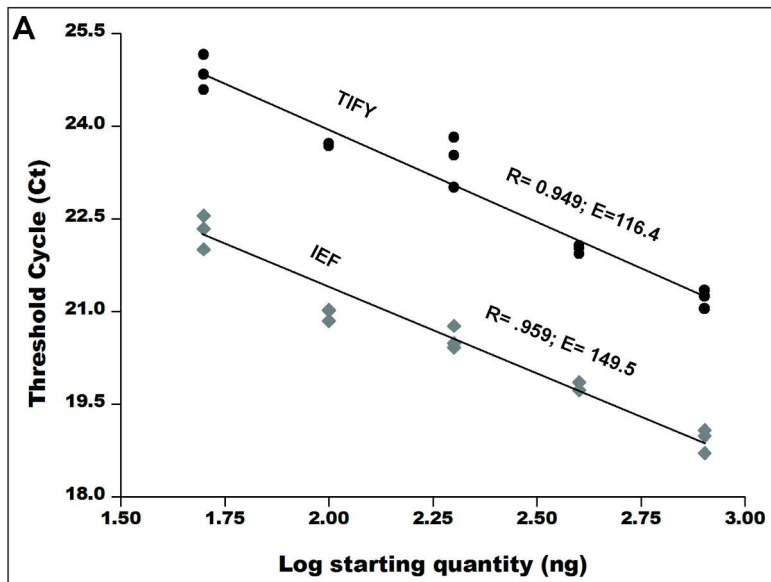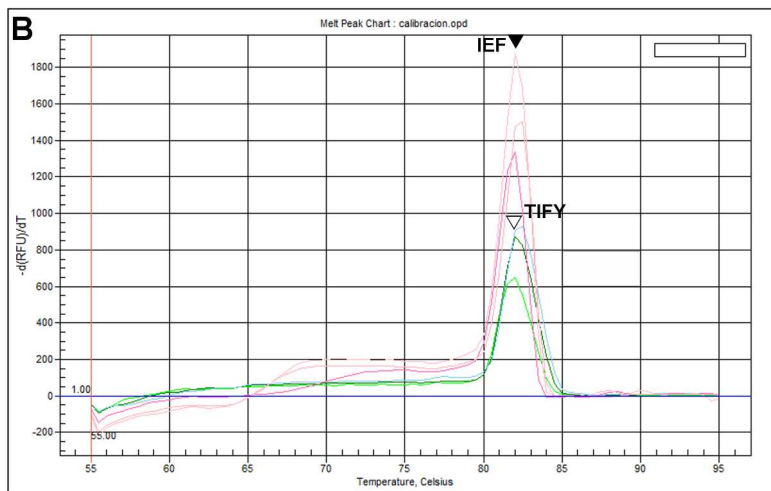

Supplement: Additional file 3 — Quality and specificity of qRT-PCR assays. A figure showing the standard curve (log starting quantity of RNA vs. Ct; upper panel) and dissociation curve (lower panel) of qRT-PCR assays of PvTIFY10C and the PvEF1 housekeeping gene used for normalization. [file 1471-2229-13-26-S3.pdf]

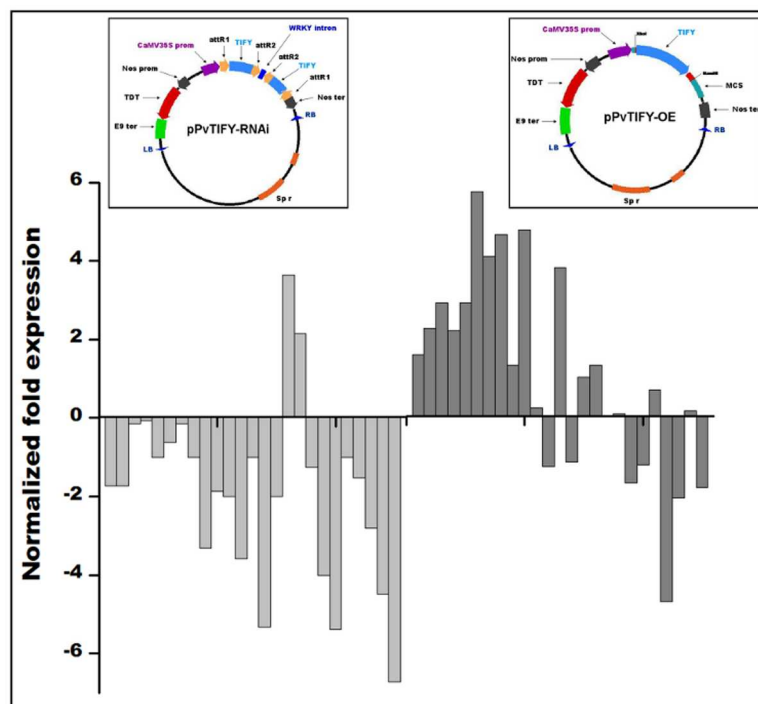

Supplement: Additional file 4 — Modulation of PvTIFY10C gene expression in transgenic roots. A figure showing diagrams representing the pPvTIFY-OE and pPvTIFY-RNAi plasmids used for PvTIFY10C over-expression and gene silencing, respectively. A graph showing the levels of PvTIFY10C expression in transgenic roots is also presented. Each bar represents the PvTIFY10C transcript level, determined by qRT-PCR, in an individual transgenic root resulting from a different transformation event with pPvTIFY-RNAi or pPvTIFY-OE. [file 1471-2229-13-26-S4.pdf]
